# Advanced MR Imaging for Knee Osteoarthritis: A Review on Local and Brain Effects

**DOI:** 10.3390/diagnostics13010054

**Published:** 2022-12-24

**Authors:** Carlo A. Mallio, Caterina Bernetti, Francesco Agostini, Massimiliano Mangone, Marco Paoloni, Gabriele Santilli, Francesca Maria Martina, Carlo C. Quattrocchi, Bruno Beomonte Zobel, Andrea Bernetti

**Affiliations:** 1 Unit of Diagnostic Imaging and Interventional Radiology, Campus Bio-Medico University of Rome, 00128 Rome, Italy; 2 Department of Anatomical and Histological Sciences, Legal Medicine and Orthopedics, Sapienza University of Rome, 00185 Rome, Italy; 3 Department of Surgical and Medical Sciences and Translational Medicine, Sapienza University of Rome, 00185 Rome, Italy

**Keywords:** knee osteoarthritis, quantitative imaging, MRI, T1ρ mapping, T2-mapping, DGEMRIC, brain

## Abstract

Knee osteoarthritis is one of the leading causes of chronic disability worldwide and is a significant social and economic burden on healthcare systems; hence it has become essential to develop methods to identify patients at risk for developing knee osteoarthritis at an early stage. Standard morphological MRI sequences are focused mostly on alterations seen in advanced stages of osteoarthritis. However, they possess low sensitivity for early, subtle, and potentially reversible changes of the degenerative process. In this review, we have summarized the state of the art with regard to innovative quantitative MRI techniques that exploit objective and quantifiable biomarkers to identify subtle alterations that occur in early stages of osteoarthritis in knee cartilage before any morphological alteration occurs and to capture potential effects on the brain. These novel MRI imaging tools are believed to have great potential for improving the current standard of care, but further research is needed to address limitations before these compositional techniques can be robustly applied in research and clinical settings.

## 1. Introduction

Knee osteoarthritis is the most common chronic joint disease and one of the leading causes of long-term disability worldwide [1,2]. Knee osteoarthritis is a slowly progressive complex musculoskeletal disorder, resulting from molecular, biochemical, and biomechanical mechanisms, supported by a multifactorial etiology, which involves genetic susceptibility and individual risk factors. It is usually an age-associated condition, with a peak prevalence in the elderly population, because there is a cumulative exposure to risk factors and biological age-related joint modifications. However, in comparison to osteoarthritis in other anatomical sites, it is usually present in earlier age groups [3,4]. Women tend to be more prone to develop it. Genetics, previous knee injuries, knee malalignment, overload or overuse of the articulation, being overweight, having muscle weakness, and joint laxity are some of the risk factors [5,6]. According to etiology, knee osteoarthritis can be classified into primary (also called idiopathic or nontraumatic, which develops without any apparent underlying reason), and secondary, whereby articular degeneration is posttraumatic or is a consequence of a pathologic basal condition with already damaged cartilage, such as in case of rheumatoid arthritis. Knee osteoarthritis usually presents with a typical symptomatic triad including (1) gradual onset of pain that worsens with activity and abates with rest, (2) stiffness in the morning or following daytime inactivity, and (3) limited range of motion and function restriction. Evaluation of standing alignment and gait is essential to reveal static varus–valgus malalignment or dynamic knee instability [1]. Even though rate of progression varies among individuals, symptoms tend to worsen over time, becoming more severe, frequent, and debilitating, eventually leading to functional impairment and disability. This condition not only alters physical functions but has also a negative impact on psychological well-being and social aspects, subsequently reducing the quality of life (QoL) [6]. Although medical history, clinical findings, and physical examination are usually sufficient for diagnosis, confirmation and severity assessment with imaging is usually necessary [7]. Multiple imaging modalities can be used to diagnose, grade, and monitor the evolution of knee osteoarthritis. Radiography and magnetic resonance imaging (MRI) are exploited most commonly [1,8,9].

A conventional plain radiograph is usually the first imaging technique requested for diagnosis and follow-up, but it is characterized by several limitations [10]. It is usually performed in a standing, flexed–fixed, weight-bearing position, at least with anteroposterior and lateral views, to assess both tibiofemoral and patellofemoral regions. It allows one to recognize predominantly morphological bone alterations, such as shape changes of the tibial plateau and femoral condyles, subchondral bone sclerosis, marginal osteophytes, or subchondral cists (geodes) formation, together with joint space narrowing. Symptom severity and pathologic imaging findings in conventional radiography are often discordant, reflecting the low sensitivity of this technique [1,8,11].

Ultrasound (US) is a highly feasible and sensitive imaging modality, without side effects, which allows for detection mainly of soft-tissue pathologies. The major limitation of US in knee joint evaluation is the possibility to partially explore the cartilage, because of acoustic windows determined by the bone [12,13].

Although MRI is considered superior for the evaluation of joints, multidetector computed tomography (CT) is more effective in the detection of bone injuries and postoperative assessment with hardware implantation [14].

Vibroarthrography (VAG) is an innovative technique that allows for articular cartilage evaluation based on both acoustic and vibrational signals generated during joint movements, which change during osteoarthritis progression. This technique enables dynamic evaluation of the joint, whereas the other diagnostic methods only provide information from a static position. VAG can be considered as a reproducible, accessible, cheap, easy in interpretation diagnostic modality, and can possibly be applied as a screening measure [15].

**Figure 1:**
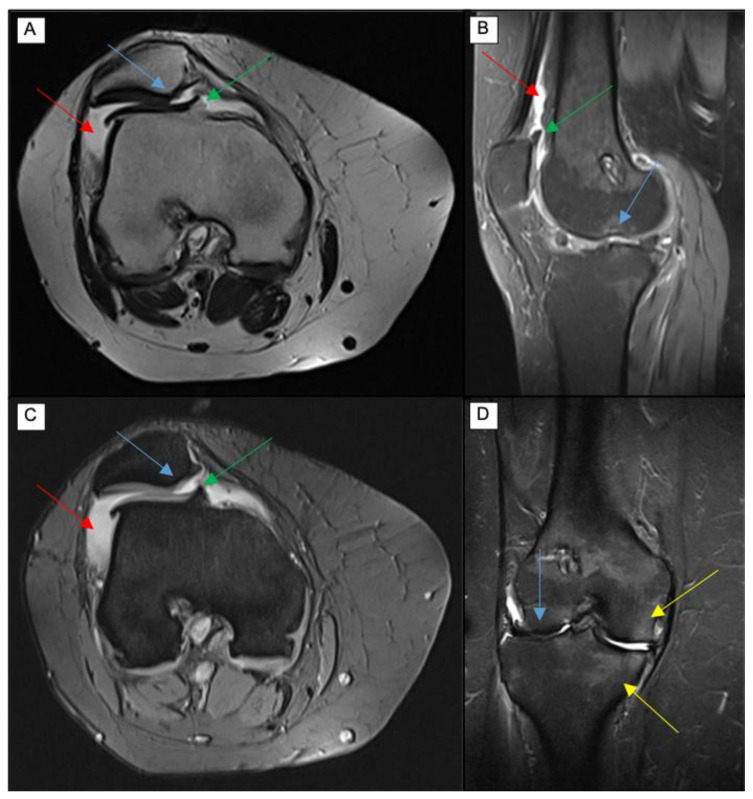
Axial T2 (**A**) and GRE (**C**), sagittal PD (**B**), and coronal STIR (**D**) MRI sequences showing features of severe knee osteoarthritis, including femoropatellar and femorotibial cartilage damage (blue arrows), effusion with synovitis (red arrows), marginal osteophytosis (green arrows), and subchondral bone marrow edema (yellow arrows).

MRI, on the other hand, is the most established imaging modality for morphological studies of the whole knee joint, to assess healthy and degenerative conditions (Figure 1). It is characterized by several advantages, including absence of radiation exposure, excellent soft tissue contrast, and the possibility of performing multiplanar and multiparametric evaluations [11]. However, it is also affected by well-known disadvantages. In particular, MR examinations are more expensive compared to X-ray or ultrasound, image acquisition is longer, noisier and more prone to artifacts limiting the diagnostic value; it is not feasible for patients with claustrophobia, there are safety hazards for patients with some metal implants and foreign bodies (e.g., pacemakers), and there is a reduced availability of MRI scans. Moreover, MRI is not able to evaluate the knee underload and the reduced visibility of deformation and mechanical axis of the limb.

Furthermore, the assessments performed with conventional MRI sequence protocols, using predominantly subjective scales and grading systems, only allow a morphological evaluation of the changes that tend to develop in the advanced stages of knee osteoarthritis [16,17].

That is why several novel MRI techniques are emerging, investigating objective and quantifiable biomarkers, able to identify biochemical alterations that occur in early stages of osteoarthritis in knee cartilage, before any morphological alteration occurs [11].

On the other hand, MRI offers a different and innovative perspective to evaluate in vivo the effects of knee osteoarthritis. Indeed, understanding the potential impact of knee osteoarthritis on brain structure and function is a fascinating topic that has been weakly investigated and will be explored further in the years to come. This approach might offer a more global view of knee osteoarthritis and will help to clarify the full impact and importance for the patient of treatments geared towards knee osteoarthritis.

The purpose of this review is to summarize these currently available compositional MRI-based techniques, underlining strengths and drawbacks. Moreover, we sought to summarize available evidence on the brain effects of knee osteoarthritis.

## 2. Hyaline Articular Cartilage of the Knee: Anatomy and Biomechanics

The knee is the joint most commonly affected by osteoarthritis [18,19]. Pathogenesis of knee osteoarthritis has been linked to biomechanical, cellular and biochemical changes, mainly regarding hyaline articular cartilage [20,21,22].

Hyaline articular cartilage has a complex structure that provides unique mechanical properties of resistance to compressive loads. It is composed of chondrocytes, type II collagen, and a hydrated matrix rich in proteoglycan, which possess water-binding properties thanks to highly negatively charged glycosaminoglycan side chains. The collagen network is the principal source of tensile and shear strength and is organized in specific zones: (1) the surface zone with tangentially aligned fibril, (2) a transitional zone with randomly aligned fibrils, and (3) a deep radial zone with fibrils aligned perpendicular to the articular surface. High glycosaminoglycan content and collagen fiber integrity are essential for the mechanical functions of healthy cartilage [21].

Early stages of osteoarthritis are hallmarked by alterations of biochemical composition in the extracellular matrix of articular cartilage, not adequately detectable with conventional MRI. A decrease in proteoglycan size and glycosaminoglycan (GAG) content, with elevation in water content and mobility, are the earliest events in the development of cartilage degeneration, accompanied immediately after, by the breakdown and disorganization of the collagen fiber network [21,22].

Cartilage degradation is progressive and irreversible because this tissue does not possess great regenerative abilities. The progression of osteoarthritis involves the whole joint, including subchondral bone, synovium, and meniscus, showing a biochemical composition similar to cartilage [23]. In fact, in more advanced stages, following the initial damage to the proteoglycan–collagen matrix, morphological changes to these tissues occur, which are evident at conventional MRI sequences [24].

Early identification of prestructural cartilage damage is essential to understand the development of cartilage degeneration, to identify preventive strategies and, also, evaluate treatments outcomes.

## 3. Role of Standard Magnetic Resonance Imaging Sequences in Knee Osteoarthritis Diagnosis

MRI is, nowadays, considered the best noninvasive and nonionizing method for morphologic assessment of knee structures. Thanks to the excellent soft-tissue contrast resolution, high spatial resolution and the possibility to perform a multiplanar and multiparametric evaluation of the knee, MRI is the most widely used technique to study the morphology of the whole joint and to identify pathological alterations, such as articular cartilage degeneration, meniscal tears, ligament abnormalities, subchondral marrow lesions, and also bone edema, synovial thickening, joint effusion, and damage to the surrounding soft tissue [11,25].

Conventional MR imaging of the knee include fast spin-echo proton-density (PD) weighted sequences, which offer excellent anatomical details and T2-weighted sequences, which help in recognition of surface defects of the cartilage, thanks to the ability to study the cartilage–synovial fluid interface, enhancing the difference of contrast between them. Eventual fat suppression sequences can be obtained to better identify marrow edema. In order to prevent overdiagnosis of cartilage lesions is necessary at least one sequence in both sagittal and coronal planes [26,27,28]. Remarkable progress has been made regarding MRI systems, with the aim of optimizing morphological cartilage imaging—for instance higher field strength, improved coils, and advanced sequences. Depending upon institutions’ software and hardware and clinical needs, additional sequences can be obtained, such as more advanced three-dimensional SE and GRE sequences (3-dimensional spoiled gradient echo, or SPGR). These volumetric sequences are indicated for a better quantification of cartilage thickness and volume and guarantee higher spatial resolution. However, disadvantages should be mentioned, such as a long scanning time, lower contrast between the cartilage and the adjacent synovial fluid, increased metallic artifact (in postsurgical knees) and uneven fat suppression. High field strength magnets (3 or 7 Tesla) are being used mainly in research settings, to improve the SNR ratio and spatial resolution with a reasonable acquisition time [27].

MR arthrography (MRA) of the knee, is not routinely used; nevertheless, it could help to increase sensitivity for chondral lesions, such as fissuring and focal or diffuse partial- or full-thickness cartilage loss, and abnormalities of the subchondral bone [18,27].

These standard morphological MRI sequences are focused mostly on alterations seen in advanced stages of osteoarthritis and possess low sensitivity for early, subtle, and potentially reversible changes of the degenerative process. Because cartilage does not regenerate and the loss is irreversible, it is crucial to capture degeneration in a prestructural stage, before morphological changes occur, with more sensitive MRI tools [29,30].

## 4. Role of Novel Compositional MR Imaging Techniques for Hyaline Knee Cartilage Evaluation

In the last decade, several quantitative MRI techniques (QMRI) are emerging as noninvasive diagnostic tools to characterize and quantify compositional changes of the hyaline articular cartilage and detect osteoarthritic changes in the knee at an early stage. These novel MR imaging techniques hold the potential to recognize biochemical, molecular, and architectural changes that happen early in the articular cartilage degeneration process, preceding significant and often irreversible, structural morphologic changes, identifiable in conventional MRI sequences or even in conventional radiography.

These imaging techniques could complement and aid standard MRI allowing to recognize the earliest and prestructural sign of osteoarthritis determined by alteration in joint homeostasis, as glycosaminoglycan depletion, reduction in water content and break-down of the collagen network [31].

Novel MRI compositional assessment techniques available for the evaluation of collagen network and proteoglycan content in the knee cartilage matrix, include relaxometry measurements (T1ρ, T2 and T2* mapping), diffusion-weighted imaging (DWI)/diffusion tensor imaging (DTI), sodium imaging, glycosaminoglycan specific chemical exchange saturation transfer (GagCEST),delayed gadolinium-enhanced MRI of cartilage (dGEMRIC), magnetization transfer, ultrashort TE (uTE), and spectroscopy [20,26,32].

Validation and implementation of these noninvasive imaging techniques that are aimed to early detect prestructural knee osteoarthritis could have a great impact in patients’ management, by providing a quantitative and objective measurements of cartilage compositional modifications, and thus, providing reliable and reproducible imaging biomarkers before irreversible morphologic changes occur [33].

Ideally, these methods can also be exploited to predict risk of the development of symptomatic and structural osteoarthritis, in order to prevent it, intervening as soon as possible to slow down the degenerative process with conservative nonpharmacological therapies, including lifestyle and behavioral intervention, such as weight loss, physical activity, or regenerative treatments, avoiding invasive surgical interventions [19,34].

However, these techniques are not widely used on a clinical routine basis due to several factors including limited availability, difficulty of acquisition with clinically feasible scan times, and the lack of standardization and validation (Table 1).

### 4.1. T1ρ Mapping

T1ρ mapping is based on the generation of T1ρ relaxation time maps, obtained by means of spin-lock MR imaging. T1ρ parameter describes the spin-lattice relaxation in the rotating frame, and it is sensitive to low-frequency, slow-motion interactions between motion-restricted water and macromolecules in the extracellular matrix. T1ρ values show a high sensitivity to proteoglycan, and hence glycosaminoglycan and water, content [21,35]. The extracellular matrix in the articular cartilage provides a motion-restricted environment to water molecules. Changes to the extracellular matrix, such as a depletion of proteoglycan/glycosaminoglycan content, which is one of the earliest signs of damaged cartilage, generally determine a higher T1ρ value [24,29,36]. Several studies suggest higher sensitivity of T1ρ than T2 mapping in the detection and measurement of cartilage degeneration, because T1ρ is highly related to decrease of glycosaminoglycan content, which is believed to be the initiating event of osteoarthritis, and due to the higher dynamic range, it can be used to detect smaller cartilage changes with a higher accuracy [21,29,37]. It appears that T1ρ could be a parameter suited to identify subjects at higher risk for developing cartilage degeneration. In particular, Stahl et al. demonstrated that T1ρ mapping could recognize different cartilage composition in asymptomatic active subjects with and without focal cartilage abnormalities. Moreover, many studies have demonstrated that T1ρ values are correlated to the severity of osteoarthritis, with higher values in advanced stages, compared to initial or intermediate stages [30,35,38]. This technique may have the potential to be used in daily clinical routine, because it does not require special preparations, contrast agent administration, or specific hardware, and the total examination time is relatively short. Nevertheless, measurements of T1ρ values necessitate an MR scanner capable of a customized and additional radiofrequency pulse sequence, leading to higher specific absorption rate (SAR) [30].

### 4.2. T2 Mapping

**Figure 2:**
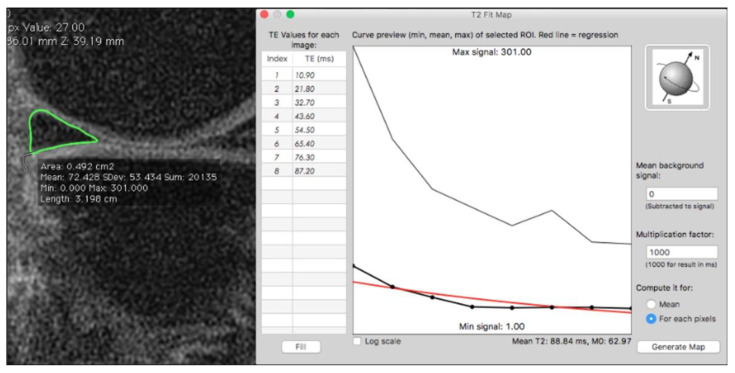
Meniscal segmentation and software graphic interface to estimate quantitative T2 mapping curve.

T2 mapping is one of the most investigated quantitative MRI methods based on generation of colored or greyscaled T2 relaxation time maps, possibly demonstrating objective early biochemical changes of the pathophysiologic processes behind cartilage degeneration (Figure 2).

T2 values describe the spin–spin relaxation time and are sensitive to the anisotropic motion of water molecules in the fibrous collagen network inside the cartilaginous matrix [21,31]. With regard to articular cartilage, T2 relaxation values are inversely related to collagen network organization and directly related to water content. Hence, the loss of collagen network integrity, along with the increment in water content and mobility, which happen in the earliest phase of cartilage degeneration, determine longer T2 relaxation times [18,21,29,32,36]. Several studies have demonstrated the potential of T2 mapping in detecting premorphologic alteration that happen in early stages of osteoarthritis and in predicting increased future risk for osteoarthritis [39,40]. However, data regarding the correlation between T2 mapping and the severity of osteoarthritis are contradictory. In particular, Dunn et al. reported a direct correlation between osteoarthritis stages and T2 values, whereas Koff et al. did not find definite differences in T2 value between early and advanced stages of osteoarthritis [21,29]. Increased T2 values are most commonly associated with cartilage damage; however, low signal-intensity lesions that may be due to increased water interaction with molecular fragments in cartilage are seen in some cases [18]. T2 maps can also be used to monitor the effectiveness of various technique of cartilage repair over time [31]. Reproducibility and validity of T2 quantification have been well documented. Indeed, longitudinal changes in T2 mapping can be achieved on most clinical MR imaging systems, as pulse sequences for obtaining quantitative T2 maps and software for generating color T2 maps are now available in commercial packages and do not require special coils [20,26,30,35].

### 4.3. T2 Star (T2*) Mapping

T2* mapping exploits T2* relaxation times to evaluate water mobility and macromolecules organization, in the cartilage. It is based on the same principle of T2 mapping; however, it is also influenced by local field inhomogeneity caused by differences in magnetic susceptibility among tissues. T2* mapping presents some advantages over conventional T2 mappings, such as higher SNR, higher spatial resolution and isotropic three dimensionality, and shorter acquisition times. However, the drawbacks include higher sensitivity to magic angle effects and susceptibility artifacts. Healthy cartilage usually shows higher T2* relaxation times compared to cartilage with early degenerative changes [34]. An interesting finding was reported in a study by Zhang et al., by measuring T2* values after long-distance running, and demonstrating a transitory increase in T2* values of knee cartilage right after a marathon, with a following reduction two months later [41]. T2* can also be used with ultrashort echo time (UTE) sequences [42].

### 4.4. Ultrashort TE

Ultrashort echo time (UTE) are MRI sequences based on low TE (<1 ms) that enable one to acquire signals from densely organized tissues characterized by very short T2 relaxation times, such as the deep calcified layers of cartilage at the osteochondral junction, highlighting tissue integrity and microarchitectural organization. The deep calcified layer where bone interface cartilage is fundamental because it is responsible for the diffusion of solute between the cartilage and the vessels, which is an important mechanism implicated in the pathogenesis of osteoarthritis and chondral repair processes. Some studies in the literature have used this sequence; for instance Ashley A Williams et al., who exploited it to demonstrate a correlation between patellofemoral deep cartilage matrix disruption after anterior cruciate ligament reconstruction (ACLR) and reduced sports and recreational activities and with gait metrics reflecting altered patellofemoral loading. According to ref. [42], uTE is known to have several issues, such as off-resonance, distortion of the slice profile and error in the radial k-space trajectories. However, off-resonance correction, efficient fat suppression and gradient calibration, could improve the results [27,42].

### 4.5. Diffusion-Weighted Imaging (DWI) and Diffusion Tensor Imaging (DTI)

Diffusion-weighted imaging (DWI) and the relative apparent diffusion coefficient (ADC) map measure water molecular diffusion, providing indirect information on collagen fiber composition and organization. Usually, healthy cartilage presents a low-ADC signal because water movement is restricted by normal collagen and proteoglycan. Conversely, degenerated cartilage with a disruption of the proteoglycan–collagen matrix, is characterized by an augmentation of water content and mobility, showing a subsequently increased ADC signal [34]. Diffusion tensor imaging (DTI) with fractional anisotropy (FA) maps, evaluate direction of water molecules within the extracellular matrix, which is related to the collagen fiber orientation and arrangement in layers. Healthy cartilage is usually characterized by anisotropic diffusion of water, with low FA values, that tend to increase in degenerated cartilage due to changes in microarchitecture. Diffusion imaging is a promising marker for demonstrating integrity of cartilage matrix, degeneration, and monitor postrepair changes. However, due to the short T2 of articular cartilage, the sensitivity to motion artifacts, the low SNR, and long scan times, more studies are necessary to achieve the resolution needed for clinical usage [30]. Ukai et al., in fact, demonstrated in a study with 41 patients, that ADC can be used to detect early-stage cartilage damage, whereas FA can also distinguish normal from damaged cartilage [43].

### 4.6. Sodium Magnetic Resonance Imaging (^23^Na+-MRI)

Sodium MRI is a validated, feasible, noninvasive MRI technique that can be used to measure proteoglycan content in articular cartilage. In sodium imaging, the ^23^Na+ cation concentration is quantified, and it is used as an indicator of proteoglycan/glycosaminoglycan content. Because sodium in cartilage is much higher than in the adjacent synovial fluid or bone, the quantitative sodium MRI has been shown to be highly specific for the glycosaminoglycan content in cartilage [24,44].

The extracellular matrix of hyaline articular cartilage contains high concentrations of proteoglycans, composed by many negatively charged glycosaminoglycan side chains, which tend to attract positively charged sodium ions (^23^Na+) to achieve electrical neutrality. Therefore, sodium and glycosaminoglycan concentrations are directly proportional [45]. In normal cartilage with abundant proteoglycan, and hence glycosaminoglycan, the sodium concentration is relatively high, whereas in early stages of damaged cartilage, which suffers of glycosaminoglycan depletion, sodium concentration tends to decrease, resulting in lower hydration, lower oncotic pressure, and collagen degradation [46]. As shown by Madelin et al., quantitative sodium MRI has the potential to detect a decrease of apparent sodium concentration (ASC) over time in articular cartilage of patients with knee osteoarthritis. The main advantage of this technique is the natural availability of ^23^Na+, which, however, presents low concentration in the tissues. Compared to proton (^1^H) imaging, sodium imaging is characterized by many limitations, such as lower resolution, lower SNR, lower gyromagnetic ratio, shorter T2 relaxation times, and higher scanning times. Further studies on hardware and software improvements, such as the use of higher magnetic field scanners, dedicated coils, and optimal pulse sequences, are needed to make sodium imaging clinically feasible [22,30].

### 4.7. Glycosaminoglycan Chemical Exchange Saturation Transfer (GagCEST)

GagCEST is a compositional MRI technique that uses specific RF pulses to identify selective saturation of hydroxyl protons of GAG, constantly transferring between GAG and water. This technique enables a quantitative assessment of GAG content in the articular cartilage matrix [22,47]. During the cartilage degeneration, GAG depletion is one of the first events and progresses during the degenerative process. Loss of GAG content determines lower GagCEST values. As demonstrated by Soellner et al., GagCEST imaging could be useful not only to identify reduction of GAG content in early osteoarthritis but could be also applied to document severity of progression. In fact, GagCEST values tend to decrease with the increase of cartilage damage [48]. Nevertheless, this technique presents many drawbacks, such as sensitivity to pH changes, a low SNR, and a difficult differentiation between hydroxyl protons and water frequency. The use of a 7T scanner could improve the performance and partially solve those issues; however, these systems are not widely available limiting clinical applicability [22,30].

### 4.8. Delayed Gadolinium-Enhanced MRI of Cartilage (dGEMRIC)

**Figure 3:**
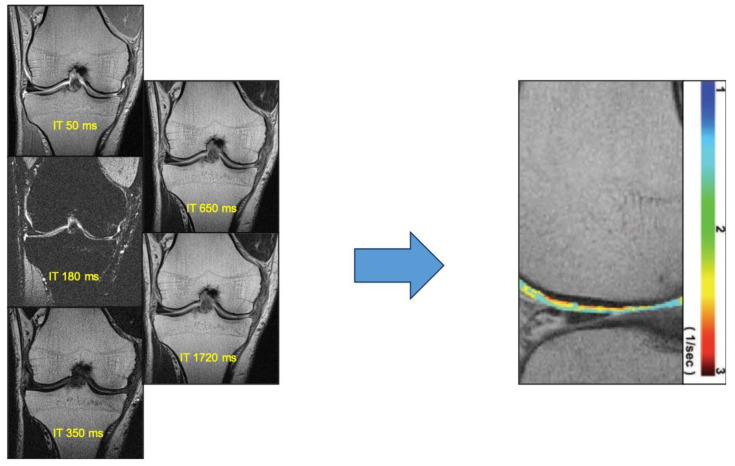
DGEMRIC technique with inversion recovery (IR) images acquired with variable inversion time (IT) and the relative color-coded map of hyaline cartilage.

dGEMRIC is based on maps of T1 values aimed to evaluate the concentration of gadolinium-based contrast agents (GBCAs) and, consequently, to indirectly quantify the GAG content (Figure 3) [49]. After GBCAs intravenous injection, the negatively charged contrast agent distributes within cartilage by diffusion, repulsing the negatively charged glycosaminoglycan side chains in the cartilage and accumulating in areas with low glycosaminoglycan concentration [50]. Therefore, GBCAs in healthy cartilage, with normal GAG concentration, will be generally low, whereas its concentration will be relatively high in damaged cartilage which is characterized by a loss of GAG. Hence, the glycosaminoglycan content is inversely proportional to the concentration of the GBCAs. Because the contrast determines acceleration of T1 relaxation times, damaged areas of cartilage, presenting higher gadolinium absorption, will present an increase of signal in postcontrast T1-weighted [22] cases. Several studies have demonstrated that dGEMRIC is a sensitive and specific technique for early identification and severity prediction of osteoarthritis. In particular, results reported in a study by Stine Hangaard et al. may suggest a point of no return for alterations in cartilage quality; however, these findings need to be addressed with larger trials [49].This method is also characterized by some disadvantages, such as the intravenous injection of GBCAs. In addition, this technique is time consuming due to required exercise and time delay of almost 90 min postcontrast injection, which are essential to allow contrast diffusion and penetration into hyaline cartilage to reach equilibration. Potential future introduction of this technique in clinical practice at 7 T will benefit from a reduction in scanning time, which can be obtained by omitting the precontrast T1 mapping acquisition [30,34].

## 5. Diagnostic Perspective: Artificial Intelligence

Artificial intelligence (AI) is one of the major recent innovations in medicine, medical imaging being one of the main fields of AI application [47,51]. It has been suggested that AI applied to knee MR images could bring faster and more accurate diagnoses and, furthermore, a better prognosis prediction of knee pathologies. Usually, the segmentation and analysis of intensity, shape, and other features of the different knee structures on MR images are performed by radiologists. However, because this approach depends on individual experience and knowledge, it usually leads to a subjective and time-consuming assessment [30]. Several studies performed in the last decade are investigating the use of AI to automatically analyze knee structures or assist radiologists, by using MR images to objectively quantify the degeneration process of knee structures. These approaches are reliable; however, user supervision is still recommended to validate the results. In the next future, it is of great interest to further explore the use of AI for analysis of knee pathologies, because it has the potential to help radiologists facing work overload, and to assist clinical decision making and reduce costs by improving indications for medical or surgical treatment [52].

## 6. Treatment of Osteoarthritis and MRI as a Tool to Follow-Up Therapies

Osteoarthritis is a progressive and degenerative condition, with unlikely regression and restoration of damaged structures. Diagnosis is often achieved in advanced stages when morphological alterations are already visible in conventional imaging. Nowadays, knee osteoarthritis management can be performed in community and primary care, with the objective to reduce pain, improve physical functional, diminish disability and improve quality of life [11,52]. Initial treatment begins with conservative methods targeted toward symptom control, in particular pharmacological (i.e., topic, oral or intraarticular medications) and nonpharmacological therapies, such as exercise and weight loss, which are proven to reduce pain and range of motion. Surgical treatment, such as partial or total joint replacement, is considered an option in severe cases, when conservative treatments fail [53]. In the last decade, there has been an improvement in the treatment options for osteoarthritis in early stages, with the aim to modify cartilage structure, like cartilage resurfacing procedures and disease-modifying drugs. These developments need to be in line with the diagnostic instruments; hence, noninvasive techniques that allow early diagnosis of osteoarthritis are necessary [29]. Conventional radiography is considered the first imaging to establish the effectiveness of disease-modifying osteoarthritis drugs by the Food and Drug Administration (FDA) [1]. MR imaging is able to detect morphologic and compositional alterations in knee cartilage; therefore, it can be useful for monitoring the effects of therapies for osteoarthritis and cartilage injury, possibly improving the indications to perform an arthroscopy or a biopsy [20,26]. Novel quantitative MRI imaging are emerging as promising noninvasive methods, for recognition of early stages of osteoarthritis, for assessing disease progression and for monitoring the restorative effects of pharmacologic or surgical therapy [30]. Validation and implementation of this new technologies in clinical setting will help to obtain early diagnosis of this disease, allowing us to make a selection of patients that could benefit from early treatment, to monitor response to therapy, to assess efforts to prevent disease progression and, ideally, to guide conservative and regenerative treatments that may prevent the progression of the degenerative process [30]. Many techniques, including dGEMRIC, have been found to be helpful in monitoring therapy and interventions, such as cartilage repair and oral medications [34]. Quantitative cartilage MRI can be applied to demonstrate structural difference after various cartilage repair strategies or between repaired cartilage and surrounding normal cartilage quantitatively and noninvasively. However, there are several inhomogeneities across studies aimed to quantitative MR evaluation after cartilage repair, such as patient cohorts, cartilage repair techniques performed, and quantitative MR imaging techniques used, with often different imaging protocols, coils, and analysis methods. The rationale and the conclusions of some of representative studies investigating these techniques are summarized in Table 2.

## 7. MRI Effects of Knee Osteoarthritis on the Brain

One of the main symptoms of knee osteoarthritis is chronic pain, which can be considered as a pain that lasts beyond the normal tissue healing time, and as a maladaptive state of pain anticipation and heightened arousal [54]. Pain in osteoarthritis is considered firstly nociceptive, provoked by damage in joint tissues; nevertheless, central mechanisms have also been recognized during pain progression and processing [37,55].

In the last decade, several studies have investigated morphometric and functional brain modification due to chronic pain; however, there are still few studies evaluating the relationship between brain structural and functional changes and knee osteoarthritis.

### 7.1. Structural Changes

One of the common structural changes reported in various chronic pain conditions is cortical grey matter volume reduction [56]. Cortical thinning could be interpreted either as an acquired feature, due to structural neuroplasticity during pain evolution with some evidence for progression or reversibility, or, alternatively, as a preexisting trait marker predisposing one to innate pain vulnerability, and unlikely to progress. In the literature, there are still discrepancies regarding the distribution of pain-related grey matter changes; in fact, the observed pattern of cortical thinning shows only partial overlap with the pain processing areas, even when studying identical primary etiologies of chronic pain disorders. This spatial dissociation may be explained by the complex multidimensionality of chronic pain experience because it can impact quality of life, reduce physical exercise, impair sleep, and possibly contribute to the various pattern of progressive cortical thinning [57,58].

Alshuft et al. reported correlation between bilateral neocortical thinning in areas extending beyond classic pain processing areas with longer duration of chronic pain independent from age and pain sensitivity in patients with knee osteoarthritis. This supports the link between acquired neocortical atrophy and chronic pain experience in knee osteoarthritis [59].

Cortical thinning with increasing duration of chronic osteoarthritis pain in the studied cohort overlapped partially with the default mode network (DMN), which is increasingly recognized as a core brain network related to homeostasis and introspection as opposed to task-oriented brain functions.

### 7.2. Functional Connectivity

Resting-state functional magnetic resonance imaging (fMRI) is a fundamental tool with which to evaluate functional connectivity (FC) of the brain, including the three main brain networks which are believed to play a dominant role in chronic pain: the default mode network (DMN), the central executive network (CEN), and the salience network (SN) [37,60,61].

DNM comprises precuneus, posterior cingulate cortex (PCC), medial frontal cortex, and lateral inferior parietal cortex. It is more active in resting states and less active during demanding cognitive tasks. This network is also involved in self-referential thoughts and memory consolidation; hence, it could be affected in patients with Alzheimer’s disease. DMN connectivity can increase with exercise [62,63]. SN is formed by bilateral anterior insula, and the dorsal anterior and anterior middle cingulate cortex. It is responsible for integrating external information, previous experiences, and a concurrent homeostatic state, in order to orient attention [64]. CEN is composed by bilateral dorsolateral prefrontal cortex and inferior parietal lobule. Both SN and CEN are active during cognitively demanding tasks [54,65].

With regards to FC, literature is still inconclusive, because some fMRI studies reported an increased FC in default mode network (DMN) in chronic pain patients, probably related to mind-wandering, whereas others found a decreased FC. DMN shows reduced brain activity during experimental pain conditions as during most other tasks. Moreover, during rest DMN FC appears to be altered in numerous chronic pain conditions including those with musculoskeletal etiology [37,66].

Cottam et al. performed a study comparing healthy controls with patients with chronic and painful knee osteoarthritis, investigating FC between the aforementioned brain areas. In symptomatic patients, the main findings were increased anticorrelation between the SN and DMN, between the right anterior insula and the PCC. Moreover, the authors reported increased negative influence between the CEN (left dorsal prefrontal cortex) and the left temporal gyrus. The right anterior insula also displayed the highest number of outflowing causal connections and the lowest number of inflowing causal connections. Huang et al. reported reduction of DMN, SN, and CEN FC in nondemented older adults with severe knee osteoarthritis treated with unilateral total knee arthroplasty under general anesthesia. These results suggest that the evaluation of preoperative cognitive functions could be useful to predict postoperative cognitive changes [66].

These findings suggest that in chronic knee osteoarthritis, there is an altered brain state at rest, with widespread static, effective, and dynamic FC changes, characterized by increased inhibitory changes, mainly related to right anterior insula, which could be considered as signs of altered pain connectome [37,54].

### 7.3. Cerebral Blood Flow

Arterial spin labeling (ASL) could be used to measure cerebral blood flow (CBF), a parameter that can be applied to evaluate potential effects of chronic pain on the brain.

A few CBF studies have investigated chronic MSK pain, reporting modifications in CBF distribution. In patients with chronic pain, Iwabuchi at al. reported increased perfusion of DMN, thalamus and sensory regions, and reduced perfusion in SN hubs, together with correlation between the fMRI and blood flow distribution [67].

ASL is a promising MR application, with a very high potential to be used in future studies to evaluate brain perfusion without usage of gadolinium-based contrast agents and to investigate the relationship with knee osteoarthritis before and after therapies.

## 8. Summary

Knee osteoarthritis is the most common form of osteoarthritis and its high prevalence rate is likely to increase in the upcoming years due to the rise of average age and weight of general population [68,69]. Because it is one of the leading causes of chronic disability worldwide, being a significant social and economic burden on healthcare systems, it is essential to develop methods to early identify patient at risk for developing knee osteoarthritis, in order to limit the progression of joint damage [70].

Conventional radiography has been the mainstay in the assessment of morphological changes in OA and continues to be fundamental for the evaluation of disease progression [10]. US can be useful for the evaluation of soft tissues in patients unable to undergo MRI or in case of patients with movement impairment, because it can also be performed bedside. CT scans can be exploited in particular cases, for instance, in the presence of bone injuries or hardware implantation [14].

VAG can be considered as a reproducible, accessible, cheap, and easily provided interpretation diagnostic modality, which can be implemented as a screening measure for patients with suspected OA [71].

However, MRI provides unparalleled visualization of the anatomical joint structures involved in the degenerative process and excellent contrast resolution [69,72,73]. Moreover, there is a growing interest toward quantitative MRI sequences that detect early biochemical microarchitecture degenerative changes occurring at the tissue level prior to structural damage. Implementation of these objective quantitative techniques will allow early diagnosis, prompt interventions, possibly preventing progression of the degenerative process, and shifting to conservative or regenerative treatments, rather than intervening surgically at the latest stages of disease [17].

In this respect, T2 relaxation time mapping and T1ρ are the most explored techniques, demonstrating validity and good reproducibility. Another option is dGEMRIC, which requires usage of GBCAs. Sodium imaging, GagCEST, and diffusion MRI are promising gadolinium-free techniques, and will be further explored.

Advancements of MRI hardware, but also improvements of image analysis tools, such as segmentation, can not only accelerate and improve repeatability of image acquisition, but also alleviate the burden of manual analysis, resolving the present logistical issues making these sequences suited for clinical settings.

AI is already being used in several fields of radiology, it is a tool with great potential that can also be exploited for analysis of knee pathologies, in order to reduce radiologists’ work overload, even though a user supervision is almost always necessary [74].

Lastly, an interesting field of study is the relationship between brain changes and knee osteoarthritis, which has been recently investigated. Brain alterations can be structural, as in the case of cortical thinning, or functional, as in the case of CBF impairment or disruption of FC and pain connectome; changes are mainly reported about the right anterior insula [62,67].

Further studies are needed to better define and understand brain abnormalities related to chronic pain and knee osteoarthritis and the potential benefits of treatments beyond the knee.

## 9. Conclusions

Novel MRI imaging tools have a great potential to improve the current standard of care, but further research is needed to address limitations before these compositional techniques can be robustly applied in research and clinical settings. Moreover, knee osteoarthritis can have effects on the brain. This is a very interesting and promising topic, which will be further explored in the future, possibly contributing to enhance the current standard of care.

## Figures and Tables

**Table 1:** Summary table of novel MRI techniques reported and their clinical feasibility.

Technique	Biochemical Changes Evaluated	Clinical Feasibility
T1ρ	PG and water content, collagen anisotropy	+++
T2	PG and water content	+++
T2 *	Macromolecule architecture and water mobility	+++
Ultrashort TE	Tissue composition and organization	+++
DWI with ADC DTI with FA	Water diffusion, tissue composition and organization	+
^23^Na-MRI	Na^+^ concentration, indirectly GAG/PG content	+
GagCEST	Exchange of hydroxyl-protons between GAG and bulk water, GAG content	+
dGEMRIC	Diffusion rate, GAG content indirectly	+

* ^23^Na-MRI, sodium magnetic resonance imaging; ADC, apparent diffusion coefficient; DTI, diffusion tensor imaging; dGEMRIC, delayed gadolinium-enhanced MRI of cartilage; DWI, diffusion-weighted imaging; FA, fractional anisotropy; GAG, glycosaminoglycan; GagCEST, GAG chemical exchange saturation transfer; MRS, magnetic resonance spectroscopy; MT, magnetization; MTR, MT ratio; PG, proteoglycan; TE, time to echo.

**Table 2:** Main characteristics of representative studies investigating novel MRI techniques for knee osteoarthritis.

Authors	Country	Aim/Rationale	Patients	MRI	Sequence	Main conclusion
H. Nishioka et al. [36]	Kumamoto, Japan	To perform qualitative evaluations of reparative tissue on articular surface of medial compartment after HCO with MRI T1ρ and T2 mapping	20	3 T scanner (Philips Healthcare, Best, The Netherlands)	T1ρ-WI T2-WI	T1ρ and T2 mapping revealed that the repaired tissue was fibrocartilage
Robert Stahl et al. [29]	San Francisco, USA	To evaluate the diagnostic value of T2 and T1ρ in identifying focal cartlage lesions in asymptomatic physically active subjects	37	3 T scanner (Signa, GE Medical Systems, Waukesha, WI)	T1ρ-WI T2-WI	T1ρ and T2 imaging demonstrated a different cartilage composition in active subjects with and without focal cartilage abnormalities
Hajimu Goto et al. [38]	Kobe, Japan	To investigate effect of aging and weight-bearing on T1ρ values in cartilage	32	3 T scanner (Philips Healthcare, Best, The Netherlands)	T1ρ-WI	The degree of weight-bearing and, in particular aging, correlate with changes in cartilage T1rho values
Timothy C. Dunn et al. [21]	San Francisco, USA	To determine differences in T2 values in femoral and tibial cartilage in patients with varying degrees of OA	55	1.5 T scanner (GE Medical Systems, Milwaukee, Wis)	T2-WI	T2 values of femoral and medial tibial cartilage increase with the severity of OA
M. F. Koff et al. [18]	Rochester, USA	To study T2 values of patellar cartilage grouped by radiographic stage of patello-femoral OA and by BMI	113	1.5 T scanner (Signa, GE Medical Systems, Waukesha, WI)	T2-WI	T2 values are not sensitive to changes in radiographic stages of OA and BMI could be considered a factor for a potential increase of T2 values
Ping Zhang et al. [41]	Shijiazhuang, China	To study effects of long-distance running on knee cartilage with T2*-WI	12	3 T scanner (Magnetom; Siemens Healthcare, Erlangen, Germany)	T2*-WI	An increase in T2* values of knee cartilage happened right after long distance running with a following reduction in the 2 months later
Ashley A. Williams et al. [42]	California, USA	To evaluate with UTE-T2* relationship between cartilage chenges, knee function, pain and gait metrics, 2 years after ACLR	60	3 T scanner (Signa, GE Medical Systems, Waukesha, WI)	UTE-T2*	Patellofemoral deep cartilage matrix disruption, as assessed by MRI UTE-T2*, was associated with reduced sports and recreational function and with gait metrics reflective of altered patellofemoral loading
Taku Ukai et al. [43]	Kanagawa, Japan	To measure damaged areas of cartilage with ADC, T2 values and FA	41	3 T scanner (Achieva 3 Tesla, Philips Healthcare, Best, The Netherlands)	ADC FA T2-WI	T2 mapping is useful for detecting moderate or severe cartilage damage. ADC can be used to detect early stage cartilage damage, FA can also distinguish normal from damaged cartilage
S. T. Soellner et al. [48]	Erlangen, Germany	To compare gagCEST of knee cartilage with intraoperative results for the assessment of early OA and to define gagCEST values for the differentiation between healthy and degenerated cartilage	21	3 T scanner (Signa, GE Medical Systems, Waukesha, WI)	gagCEST	gagCEST might provide a diagnostic tool for the detection of early knee-joint cartilage damage and grading
Stine Hangaard et al. [49]	Copenhagen, Denmark	To evaluate changes in quality of cartilage after weight loss	19	1.5 T scanner (Philips Healthcare, Best, The Netherlands)	dGEMRIC	Improvement of cartilage quality, assessed with dGEMRIC, after weight loss might be possible only in early stage of KOA
Guillaume Madelin et al. [46]	New York, USA	To evaluate the potential of sodium MRI to detect changes over time of apparent sodium concentration (ASC) in articular cartilage in patients with KOA	12	7 T scanner (Siemens Healthcare, Erlangen, Germany)	^23^Na^+^-MRI	Quantitative sodium MRI has the potential to detect a decrease of ASC over time in articular cartilage of patients with KOA

^23^Na-MRI, sodium magnetic resonance imaging; ADC, apparent diffusion coefficient; DTI, diffusion tensor imaging; BMI, body mass index; dGEMRIC, delayed gadolinium-enhanced MRI of cartilage; DWI, diffusion-weighted imaging; FA, fractional anisotropy; GAG, glycosaminoglycan; GagCEST, GAG chemical exchange saturation transfer; HCO, hemicallotasis osteotomy; KOA, knee osteoarthritis; MRS, magnetic resonance spectroscopy; MTR, magnetization ratio; OA, osteoarthritis; TE, time to echo; UTE, ultrashort echo time; WI, weighted imaging.

## Data Availability

Not applicable.
